# Abrogation of Cellular Senescence Induced by Temozolomide in Glioblastoma Cells: Search for Senolytics

**DOI:** 10.3390/cells11162588

**Published:** 2022-08-19

**Authors:** Lea Beltzig, Markus Christmann, Bernd Kaina

**Affiliations:** Institute of Toxicology, University Medical Center Mainz, Obere Zahlbacher Str. 67, D-55131 Mainz, Germany

**Keywords:** cellular senescence, senolytics, glioma, temozolomide, cell death, apoptosis, fisetin, artesunate, chloroquine, curcumin

## Abstract

A first-line therapeutic for high-grade glioma, notably glioblastoma (GBM), is the DNA methylating drug temozolomide (TMZ). Previously, we showed that TMZ induces not only apoptosis and autophagy, but also cellular senescence (CSEN). We presented the hypothesis that GBM cells may escape from CSEN, giving rise to recurrent tumors. Furthermore, the inflammatory phenotype associated with CSEN may attenuate chemotherapy and drive tumor progression. Therefore, treatments that specifically target senescent cells, i.e., senolytic drugs, may lead to a better outcome of GBM therapy by preventing recurrences and tumor inflammation. Here, we tested Bcl-2 targeting drugs including ABT-737, ABT-263 (navitoclax), several natural substances such as artesunate, fisetin and curcumin as well as lomustine (CCNU) and ionizing radiation (IR) for their senolytic capacity in GBM cells. Additionally, several proteins involved in the DNA damage response (DDR), ATM, ATR, Chk1/2, p53, p21, NF-kB, Rad51, PARP, IAPs and autophagy, a pathway involved in CSEN induction, were tested for their impact in maintaining CSEN. Treatment of GBM cells with a low dose of TMZ for 8–10 days resulted in >80% CSEN, confirming CSEN to be the major trait induced by TMZ. To identify senolytics, we treated the senescent population with the compounds of interest and found that ABT-737, navitoclax, chloroquine, ATMi, ATRi, BV-6, PX-866 and the natural compounds fisetin and artesunate exhibit senolytic activity, inducing death in senescent cells more efficiently than in proliferating cells. Curcumin showed the opposite effect. No specific effect on CSEN cells was observed by inhibition of Chk1/Chk2, p21, NF-kB, Rad51 and PARP. We conclude that these factors neither play a critical role in maintaining TMZ-induced CSEN nor can their inhibitors be considered as senolytics. Since IR and CCNU did not exhibit senolytic activity, radio- and chemotherapy with alkylating drugs is not designed to eliminate TMZ-induced senescent cancer cells.

## 1. Introduction

Despite tremendous progress in alternative therapies, genotoxic drugs still play a key role in the treatment of many cancers. They exert their cytotoxic effect through a variety of toxic mechanisms such as DNA alkylation, DNA crosslinking, inhibition of DNA ligation, and insertion of poisoned bases, all of which are aimed to destroy cancer cells and spare the healthy tissue [1]. Cell death due to DNA damage is mainly executed through apoptosis [2]. However, over the past years, several studies demonstrated that not only cell death, but also senescence is a central outcome of cancer radio- and chemotherapy [3]. Since senescent cells do not proliferate, senescence has long been an accepted and wishful side effect of cancer therapy and thought to be not disadvantageous for the patient. However, over the past few years, several studies demonstrated that senescent cells are not absolutely irreversibly arrested, but might escape from cell cycle arrest and start proliferating again, posing a threat to the patient as senescent cells often are resistant to the therapy they escaped from [4]. Additionally, cellular senescence (CSEN) is characterized by the senescence associated secretory phenotype (SASP) [5], i.e., they secrete several inflammatory components, causing chemotherapy side effects and local inflammation, which is a driving force of tumor progression [6]. Therefore, a strategy to improve the patient’s response to senescence-inducing cancer therapies might be the administration of senolytic drugs following radio-chemotherapy. Senolytic drugs are compounds that specifically kill (therapy-induced) senescent, but not proliferating non-senescent cells [7].

Our work focuses on glioblastoma (GBM), a high-grade (WHO grade 4) glioma with a dismal prognosis of about 15 months following diagnosis, despite treatments that involve resection of the tumor, chemo- and radiotherapy [8]. The main chemotherapeutic used for GBM treatment is the alkylating agent temozolomide (TMZ), which gives rise to several N- and O-methylated DNA adducts [9,10]. Our and other laboratories demonstrated that the minor lesion O^6^-methylguanine (O^6^MeG) is the key driver of TMZ-induced cell responses by inducing DNA double-strand breaks (DSBs) via futile mismatch repair (MMR) cycles [11]. Further experiments showed that these DSBs induce the DNA damage response (DDR) pathway through activation of the ATM/ATR-p53-p21 and SIAH1-HIPK2 axis [12], triggering mainly (>80%) senescence and only a little (<30%) apoptosis [13]. Cells phenotypically resembling senescent cells were also found in GBM specimens, with higher frequency in recurrences than in the primary tumor [13]. It is conceivable that the high level of senescent cells resulting from TMZ therapy gives rise to recurrent tumors exhibiting the SASP and a poor response to TMZ and other anticancer drugs used in the recurrent situation, which might explain the dismal prognosis of the disease. Based on this, it is pertinent to hypothesize that elimination of therapy-induced senescent cells might result in a better overall and progression free survival. Therefore, the identification of senolytic drugs that kill senescent GBM cells following radio-chemotherapy with TMZ treatment is a worthwhile task. 

To this end, we screened natural substances and several small compounds for their senolytic capacity. Ionizing radiation (IR) and lomustine (CCNU) were included for comparison. Senescent glioblastoma cells were generated upon TMZ exposure and treated with the compounds of interest in concentrations that are non-toxic on proliferating cells. A significant reduction of the senescence fraction accompanied by apoptotic cell death was observed following treatment with some natural compounds (fisetin, artesunate), chloroquine, ATR and ATM inhibitors and small molecule inhibitors of cIAP1/XIAP and Bcl-2. Since these compounds killed CSEN more efficiently than proliferating cells, they can be considered senolytics. No specific effect on CSEN was seen when cells were treated with downstream inhibitors of DDR, autophagy as well as IR and CCNU, which obviously do not bear senolytic activity.

## 2. Material and Methods

### 2.1. Cell Lines and Culture Conditions

The two human glioblastoma cell lines LN229 and A172, obtained from American Type Culture Collection (ATCC), were cultured in 10 % fetal calf serum and GlutaMax containing DMEM (Gibco, Life Technologies Corporation, Carlsbad, USA. They were kept in a humidified 5% CO_2_ atmosphere at 37 °C. For apoptosis and senescence experiments, cells were seeded at a density of 1 × 10^5^ cells per 5-cm or 6-well dish, for MTT Assay, cells were seeded in 96-well plates at a density of 5 × 10^3^ cells/well and for collecting lysates, cells were seeded in 10 cm dishes at a density of 2 × 10^5^ cells/dish. Cells were treated when they were in the exponential growth phase. 

### 2.2. Drugs and Treatment Conditions

TMZ was obtained from Dr. Geoff Margison (University of Manchester, Manchester, UK). The 150 mM stock solution was made by dissolving TMZ in DMSO, kept at −80 °C and further diluted (1:10) in sterile distilled water directly before addition to the medium at the desired final concentration. Thereby, the DMSO concentration during the treatment was kept at a minimum. Native curcumin (CAS 458-37-7, Sigma Aldrich, Steinheim, Germany) and fisetin (CAS 528-48-3, Abcam plc., Cambridge, UK) were dissolved in ethanol and stored as 10 mM stock. Micellar curcumin, kindly provided by AQUANOVA AG (Darmstadt, Germany), was freshly prepared in PBS. Artesunate (ProJect Pharmaceuticals GmbH, Martinsried, Germany) was dissolved in DMSO at a stock concentration of 20 mg/mL. The inhibitors for ATM, ATR, Chk1, Chk2, NF-kB and p21 were prepared, stored, and used as previously described [14]. The p53 inhibitors pifithrin-a (CAS 63208-82-2, Selleck Chemicals, Houston, USA), and pifithrin-µ (CAS 64984-31-2, Selleck Chemicals, Houston, USA) were dissolved in DMSO and used at a final concentration of 10 µM. The PI3K inhibitor PX866 (CAS 502632-66-8, Cell Signaling Technology Inc., Cambridge, UK), the nociceptin inhibitor MCOPPB trihydrochloride (CAS 108147-88-1, Selleck Chemicals), the cIAP1/XIAP inhibitor BV6 (CAS 1001600-56-1, Selleck Chemicals, Houston, TX, USA) and the Rad51 inhibitor Ri1 (CAS 415713-60-9, Axon Medchem, Groningen, NL, USA) were dissolved in DMSO and used at final concentrations of 1 µM, 2.5 µM and 5 µM, respectively. The PARP inhibitor veliparib (CAS 912444-00-9, Axon Medchem, Groningen, NL) was dissolved in ethanol and used at a final concentration of 10 µM. The autophagy inhibitors 3-MeA (CAS 5142-23-4, Sigma-Aldrich, Steinheim, Germany) and chloroquine (CAS 54-05-7, Sigma-Aldrich, Steinheim, Germany) were dissolved in DMSO and sterile distilled water, respectively, at 200 mM and 50 mM stock concentrations, respectively. All inhibitors were kept at −20 °C, divided into aliquots to avoid freeze–thaw cycles.

### 2.3. MTT Assay

Analysis of cell viability via the MTT assay was performed as previously described [15]. In brief, cells were washed with PBS and incubated with 0.5 mg/mL MTT (Biomol, Germany) reagent in DMEM without phenol red (Gibco, Life Technologies Corporation, UK) for 2 h in the incubator. The staining solution was thereafter discarded, and cells were incubated with 0.04 N HCl on a shaker at RT for 10 min. Staining was measured at OD_570_, using the Berthold microplate reader. The absorbance of treated cells relative to proliferating cells was used as a measure of cell viability. 

### 2.4. Quantification of Apoptosis and Senescence

Apoptosis and senescence were analyzed by flow cytometry as described previously [16]. In brief, for apoptosis measurement, cells including the supernatant were collected by trypsinization, resuspended in annexin-binding buffer containing 2.5 µL annexin-FITC (Miltenyi Biotec, Bergisch-Gladbach, Germany) and incubated for 15 min at RT. Thereafter, 10 µL of a 50 µg/mL propidium iodide (PI) solution were added to the samples and cells were stored on ice until measurement. For quantification of senescence, cells were pre-incubated with 300 µM chloroquine for 30 min in the incubator to inhibit endogenous β-galactosidase (β-gal) activity. Cells were then incubated with C_12_FDG (Abcam, Cambridhe, UK) for an additional 90 min. Cells without supernatant were collected by trypsinization, resuspended in PBS, and stored on ice until measurement. For both analyses, cells were kept in the dark from substrate addition until measurement with the FACS Canto II flow cytometer. Proliferating cells were used as controls. Data were analyzed using the Flowing Software 2 program (Turku Center for Biotechnology, University of Turku, Finland). 

## 3. Results

### 3.1. Senolytic Activity of ABT-737 and ABT-263 in GBM Cells

**Figure 1:**
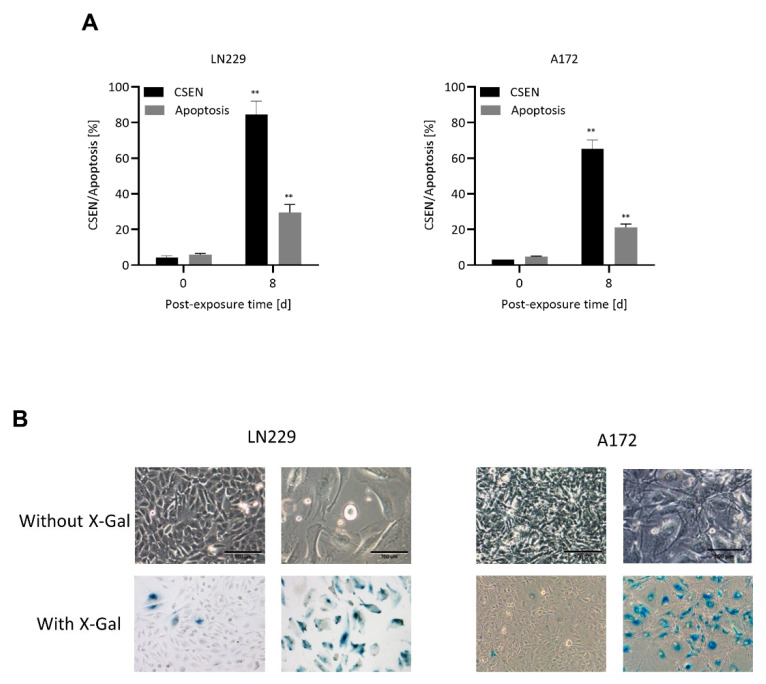
Induction of apoptosis and senescence. (**A**) Proliferating (day 0) and TMZ (50 µM) treated LN229 and A172 cells (day 8) were measured for apoptosis and senescence via annexin/PI and C_12_FDG staining, respectively. TMZ treated cells display high amounts of senescence (CSEN) and low apoptosis levels. (**B**) Exemplary images of proliferating (left) and senescent (right) LN229 and A172 cells showing enlarged, flat morphology and SA-β-gal staining. Data in (**A**) represent the mean of 3 independent experiments ± SEM. ** *p* < 0.01.

A quantification of the senescent population obtained 8 d after TMZ treatment is shown in Figure 1A, confirming that senescence is the main trait induced by TMZ in MGMT lacking GBM cells, amounting up to 80% at day > 8 following treatment, whereas apoptosis is a minor trait with only 20–30%. The senescent population, which exhibited the key properties of cellular senescence (CSEN) including SA-ß-GAL staining, enlarged size and flat morphology (representative images in Figure 1B and Supplementary Figure S1), was used for screening of the senolytic potential of different drugs, natural compounds, CCNU and IR.

**Figure 2:**
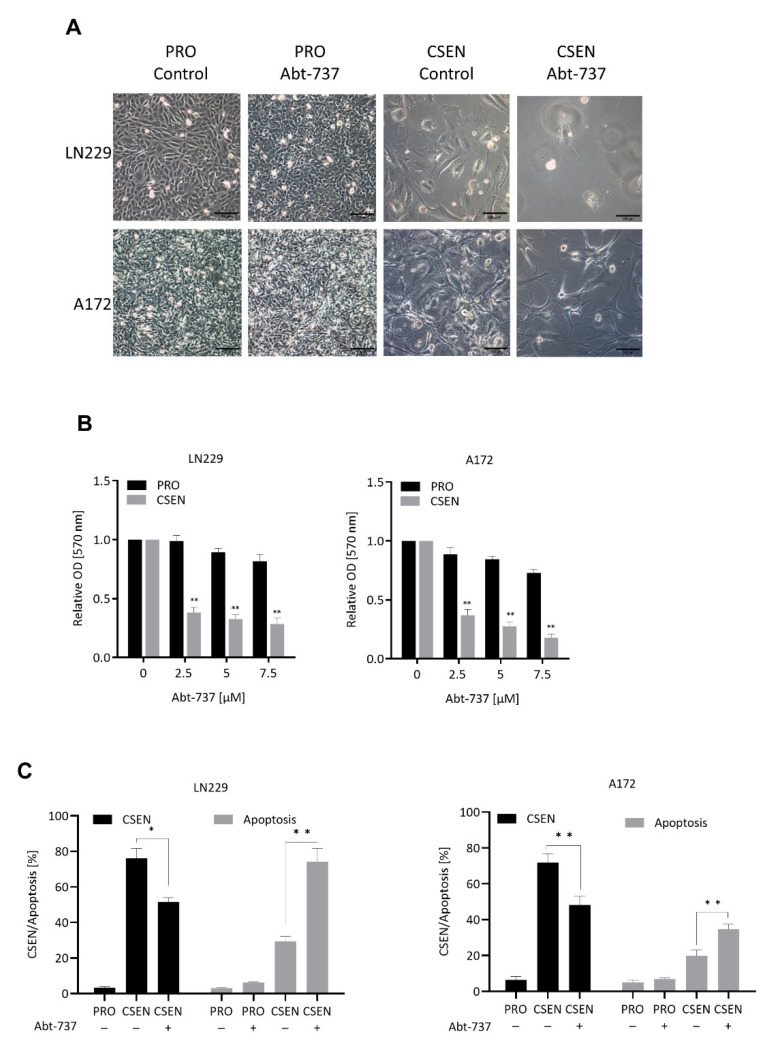
Effect of ABT-737 on senescent cells. (**A**) Representative images of proliferating (PRO) and senescent (CSEN) LN229 and A172 cells without (control) and with 2.5 µM ABT-737 treatment. (**B**) Treatment of senescent LN229 and A172 cells with increasing concentrations of ABT-737 leads to a significant decrease in cell viability, as shown by MTT assay. (**C**) Decrease in senescence and increase in apoptosis shown by flow cytometry when senescent LN229 and A172 cells were treated with a non-toxic concentration (2.5 µM) of Abt-737. Data in (**B**,**C**) represent the mean of >3 independent experiments ± SEM. * *p* < 0.05, ** *p* < 0.01.

First, we tested a well-established senolytic drug, ABT-737 [17], and treated senescent LN229 and A172 cells with rising concentrations, starting at 2.5 µM. Two days following treatment, senescent cells showed signs of cell death already with the lowest applied dose of ABT-737, while proliferating cells did not (Figure 2A for morphologic images). Senolytic drugs are defined as being able to kill senescent, but not proliferating cells. Analysis of cell viability by MTT assay demonstrated a significant dose-dependent decrease in the senescent, but not exponentially growing population (Figure 2B). A concentration of 2.5 µM ABT-737 was non-toxic on proliferating LN229 and A172 cells (Figure 2B,C) and, therefore, chosen for further quantitative analysis by C_12_FDG flow cytometry. Treating TMZ-induced senescent cells with this dose, we observed a significant decrease of CSEN in the population. This was accompanied by an increase in apoptosis in both cell lines (Figure 2C). The data clearly demonstrate that ABT-737 has senolytic activity, which is detectable two days following treatment. Based on this data, we treated TMZ-induced CSEN cells in further experiments for two days with the agents to be tested, administering non-toxic/sub-toxic concentrations (Supplementary Figure S2).

**Figure 3:**
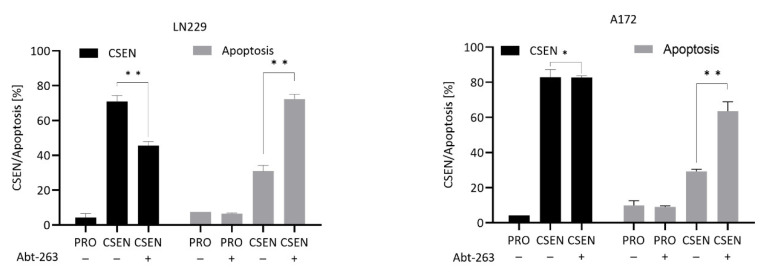
Effect of ABT-263 on senescent cells. Treatment of senescent LN229 cells with ABT-263 leads to a significant decrease in senescence and an increase in apoptosis. In A172 cells, reduction in senescence was not observed, while apoptosis increased significantly. PRO, proliferating cells; CSEN, senescent cells. Data represent mean of >3 independent experiments ± SEM. * *p* < 0.05, ** *p* < 0.01.

A synthetic drug acting similar to ABT-737 is ABT-263 (navitoclax), which has already entered clinical trials [18]. As shown in Figure 3, ABT-263 in a non-toxic concentration reduced CSEN and increased the apoptosis level in the senescent population, indicating the compound bears senolytic activity on GBM cells.

### 3.2. Screening of Diverse Compounds for Their Senolytic Activity

**Figure 4:**
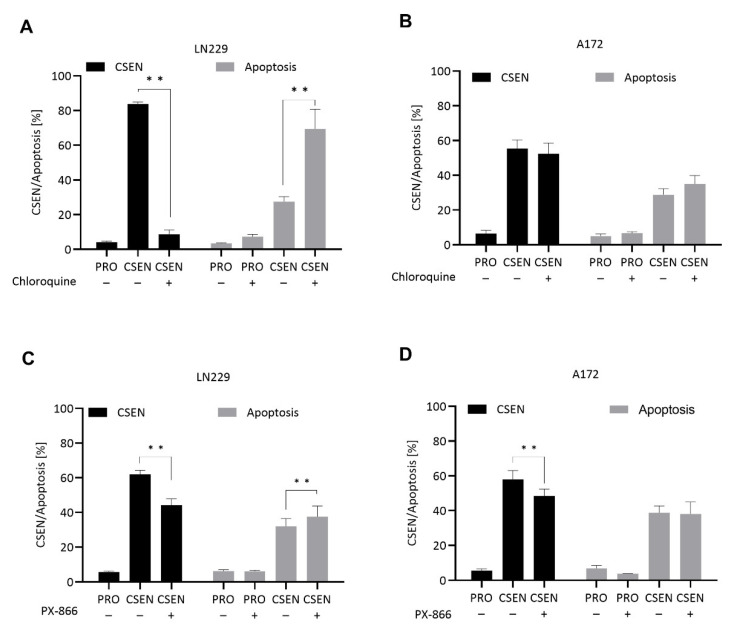
Identification of autophagy inhibitors as senolytic compounds. Proliferating (PRO) and senescent (CSEN) LN229 and A172 cells were treated with several substances to test their senolytic capacity. A significant decrease in senescence was observed by chloroquine (20 µM) in LN229 cells (**A**) as well as PX866 in LN229 and A172 cells (**C,D**). Both substances induced apoptosis in senescent LN229 (**A,C**), but not A172 (**B,D**) cells. Data represent the mean of >3 independent experiments ± SEM. ** *p* < 0.01.

Treatment of senescent LN229 cells with the autophagy inhibiting drugs chloroquine and PX-866 caused a significant decline in the CSEN fraction accompanied by an increase in apoptosis (Figure 4A,C). In A172 cells, chloroquine was ineffective (Figure 4B), while PX866 reduced the CSEN level (Figure 4D). This indicates a senolytic effect of chloroquine (20 µM) in LN229, but not A172 cells. Even though a reduction in senescence was not necessarily accompanied by an increase in apoptosis, which might be due to early clearance of apoptotic cells, the results indicate that the autophagy inhibitors bear a senolytic potential, at least in LN-229 cells.

**Figure 5:**
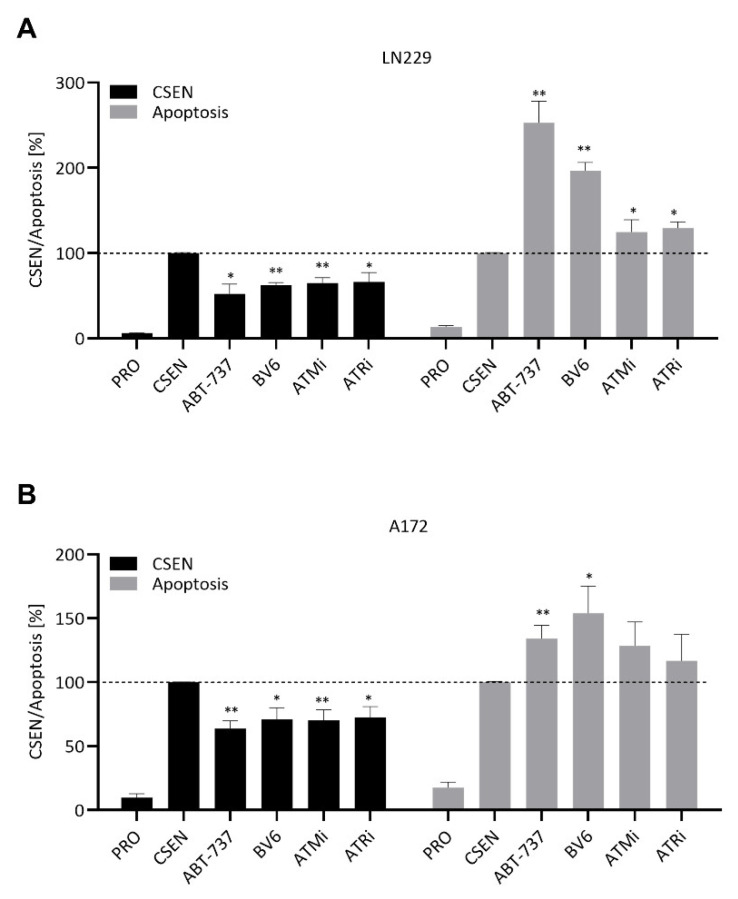
Identification of diverse compounds with senolytic activity. Proliferating (PRO) and senescent (CSEN) LN229 and A172 cells were treated with several substances to test their senolytic capacity. ABT-737 served as positive control. Inhibition of cIAP1/XIAP (BV6), ATM and ATR reduced senescence levels in LN229 (**A**) and A172 (**B**) cells. Apoptosis was concomitantly increased for all substance in LN229 (**A**), while only inhibition of cIAP1/XIAP had such effect in A172s cells (**B**). Data represent mean of >3 independent experiments ± SEM. * *p* < 0.05, ** *p* < 0.01.

To broaden our screen for senolytic drugs, we treated senescent cells with several inhibitors of apoptosis and the DNA damage response. Targeting the inhibitors of apoptosis cIAP1/XIAP with BV6 led to a significant reduction in senescence levels in LN229 and A172 cells concomitant with an increase in apoptosis (Figure 5A,B). 

Since CSEN cells exhibit a high level of DSBs and sustained DDR activation, we inhibited ATM and ATR, which are the upstream kinases activated by DSBs. Interestingly, inhibition of either ATM (with AZD1390) or ATR (with VE-821) led to a significant reduction in CSEN levels in both cell lines (Figure 5A,B). A significant increase in apoptosis was observed in LN229 cells for all three inhibitors (Figure 5A), while an increase in apoptosis in senescent A172 cells was observed only for BV6 (Figure 5B).

**Figure 6:**
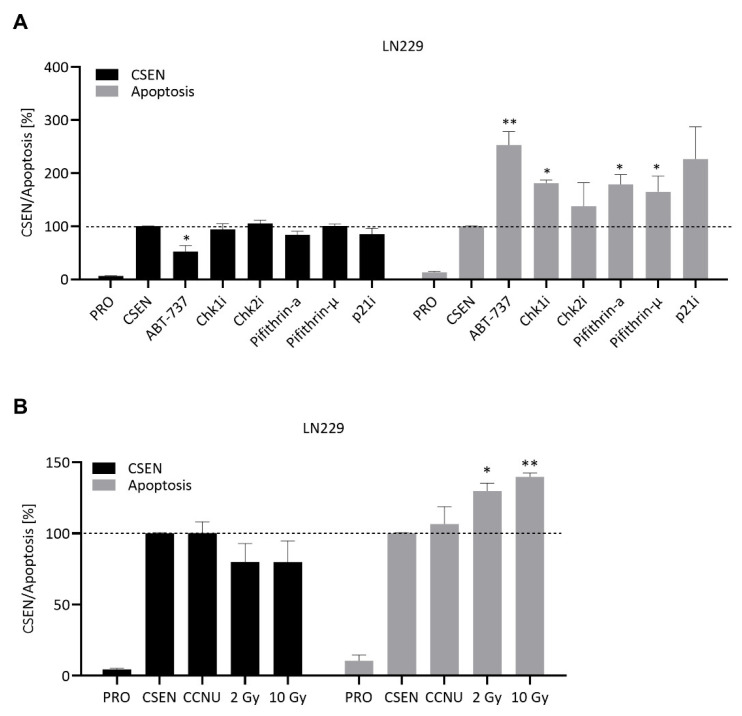
Search for senolytics. Proliferating (PRO) and senescent (CSEN) LN229 and A172 cells were treated with several substances. ABT-737 served as positive control. No senolytic effect was observed when LN229 were treated with the corresponding inhibitors for Chk1/2, p53 or p21 (**A**). A senolytic effect was also not observed upon treatment with CCNU or radiation (**B**). Data represent the mean of >3 independent experiments ± SEM. * *p* < 0.05, ** *p* < 0.01.

Since ATM and ATR inhibition provoked a senolytic effect, we assessed the inhibition of their downstream-targets Chk1/2, p53 and p21. Although the inhibitors were slightly toxic on proliferating and CSEN cells, no senolytic activity was observed (Figure 6A). The same was true for inhibition of NF-kB, Rad51, PARP and the nociceptin receptor (Figure S3A). We also analyzed the effect of the autophagy inhibitor 3-MeA on senescent cells. Senescence and apoptosis levels remained stable following treatment of both LN229 and A172 cells (Figure S3A,B). Therefore, we conclude that these small molecule inhibitors exhibit no senolytic activity on GBM cells.

### 3.3. CCNU and IR Are Not Senolytic

Besides TMZ, the chloroethylating drug CCNU is frequently used (2nd line) in GBM therapy. To test if DNA damage induced by CCNU might lead to the death of senescent cells, we treated senescent LN229 and A172 cells with 10 µM CCNU, a concentration non-toxic on proliferating cells (Figure S2A,B). No reduction in senescence levels was observed (Figure 6B). Hence, CCNU cannot be considered as a senolytic drug. To analyze the effect of IR on senescent cells, they were irradiated with doses of 2 and 10 Gy. A slight non-significant reduction in senescence was observed together with an increase in apoptosis level (Figure 6B). Since IR is toxic on both replicating (Figure S2C) and senescent cells (Figure 6B), it cannot be classified as senolytic. 

### 3.4. Natural Compounds Bearing Senolytic Capacity

**Figure 7:**
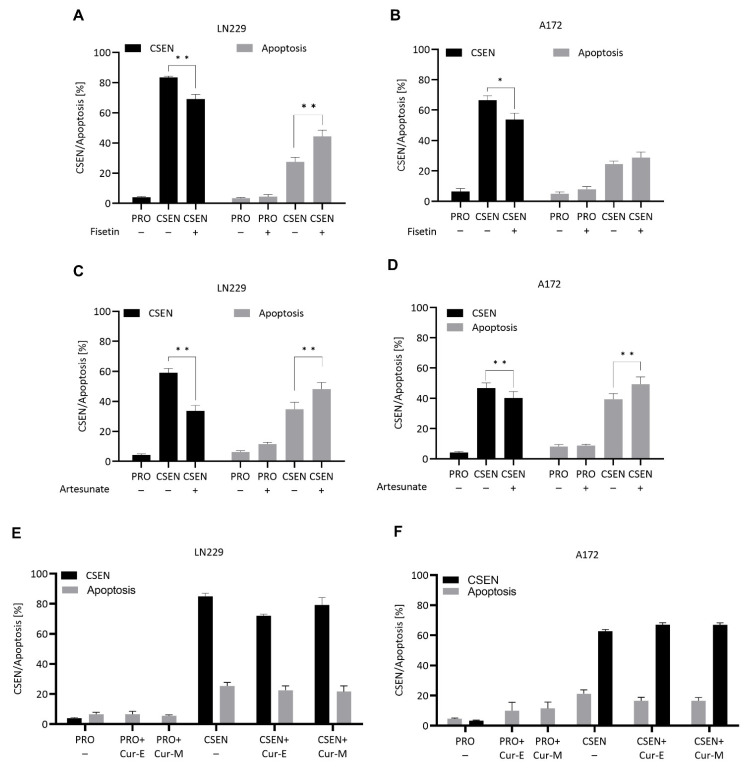
Effect of natural compounds on senescent cells. Treatment of proliferating (PRO) and senescent (CSEN) LN229 and A172 cells with fisetin (**A**,**B**) and artesunate (**C**,**D**) leads to a significant decrease in senescence and increase in apoptosis, while treatment with curcumin (10 µM) was without effect (**E**,**F**). Cur-E, curcumin solubilized in ethanol; Cur-M, curcumin embedded in micelles. Without treatment (−). Data represent the mean of >3 independent experiments ± SEM. * *p* < 0.05, ** *p* < 0.01.

To identify senolytic agents, we investigated the effect of three natural compounds on TMZ-induced senescent GBM cells: fisetin, artesunate and curcumin. Fisetin (20 µM) was not toxic on proliferating cells (Figure S2A,B), while analysis of senescence showed a significant reduction in the senescence levels in LN229 (Figure 7A) and A172 cells (Figure 7B). The data clearly shows that fisetin has senolytic activity in GBM cells. 

Artesunate is a pharmacologically active ingredient of *Artemisia annua* [19]. With a concentration of 15 µM, artesunate was not toxic on proliferating cells, while treatment of senescent LN229 and A172 cells led to a significant reduction of senescent cells and simultaneous increase in apoptosis in LN229 and A172 cells (Figure 7C,D), indicating that artesunate has senolytic activity in GBM cells. 

We also tested the senolytic activity of curcumin. We observed neither a reduction in senescence nor an induction of apoptosis following treatment of senescent cells with a subtoxic concentration of 10 µM curcumin (Figure 7E,F).

## 4. Discussion

Previously, we reported that senescence is the main cellular response following TMZ treatment of glioblastoma cells [13,14,20], and studies on glioblastoma radiation responses revealed that ionizing radiation is also a potent inducer of CSEN [21]. Since the primary therapy of glioblastoma rests on TMZ and radiation, CSEN is likely the main outcome of adjuvant GBM radio-chemotherapy. It has been hypothesized that senescent cells may escape division arrest, re-starting proliferation, which gained some experimental support [22,23]. Additionally, senescent cells exhibit the senescence-associated inflammatory phenotype (SAIP) that is characterized by secretion of proinflammatory cytokines, which can promote tumor progression, leading to adverse chemotherapeutic effects [6]. Therefore, eliminating therapy-induced senescent cancer cells is a desirable approach, and understanding of senescence maintenance and the identification of senolytic agents is an important issue in cancer therapy.

### 4.1. BCL Inhibitors

In the present study, we defined a senolytic as an agent that reduces the number of CSEN cells in a senescent population. This can occur through induction of apoptosis, but other pathways may also be evoked in CSEN cells. In our experiments, the senescent population was obtained by TMZ treatment followed by 8–10 d recovery, which yielded >80% senescent cells (for time course see ref. [13]). For identification of senolytic compounds, we measured senescence and apoptosis simultaneously. As reference compound we used ABT-737. This small molecule has been proven in several in vitro and in vivo models to be an effective senolytic agent. By selectively targeting Bcl-2, Bcl-W and Bcl-XL, it interrupts the anti-apoptotic pathway and induces apoptosis in senescent cells [17,24]. To show that ABT-737 is senolytic in our glioma cell system, we treated TMZ-induced senescent LN229 and A172 cells with ABT-737 for 2 days. Quantitative analysis clearly showed a significant reduction of senescent cells, while concomitantly apoptosis was significantly enhanced, revealing that ABT-737 has senolytic activity in our glioma cell system. It is important to note that the same concentration of ABT-737 was without toxic effect on the corresponding proliferating cell populations. This confirmed that ABT-737 can be used as a positive control in our experiments. 

Several other BH3-mimetic compounds have been developed, including ABT-263 (navitoclax). Like ABT-737, the compound is a selective inhibitor of Bcl-2 and Bcl-X_L_ and exhibits high senolytic activity in several in vitro and in vivo model systems [17,25,26]. It is being used already for clinical studies [18]. To show that ABT-263 also exerts senolytic activity on GBM cells, we treated senescent LN229 and A172 with the drug. Again, a reduction in the senescence level was observed and concomitantly cell death was significantly increased. We should note that reduction in senescence was not always associated with the same quantitative increase in apoptosis as early stages of apoptosis may still exhibit the senescence marker (C_12_FDG). Having established the experimental system, we tested several compounds for their ability to kill selectively senescent GBM cells, including inhibitors of the DDR and autophagy, IR, CCNU and natural compounds. 

### 4.2. IAP Inhibitor

Since senescence often rests on inhibition of apoptosis, it is reasonable to suppose that activation of apoptosis-inhibiting factors provokes a senolytic response. We previously reported that upon TMZ treatment, the anti-apoptosis factor c-IAP2 is upregulated and that its inhibition by the smac mimetic BV6 in the TMZ post-treatment period leads to an increase in cell death [27], indicating the inhibitor is effective in the CSEN induction phase. To study whether BV6 is effective in the CSEN maintenance phase, we treated senescent populations of LN229 and A172. Interestingly, the measurements showed a decrease in the senescent population, while apoptosis concomitantly increased. Obviously, BV6 acts as a senolytic agent in glioma cells, which was not reported before.

### 4.3. Autophagy Inhibitors

Chloroquine has been used over decades for malaria treatment until resistant populations developed. These days, the aminoquinoline is used for the treatment of rheumatoid arthritis and as it acts as a mild immunosuppressant and anti-inflammatory drug [28]. Chloroquine is an inhibitor of the late stage of autophagy, by decreasing the lysosomal function through blocking the fusion of lysosomes and autophagosome formation [29,30]. The inhibitory effect on autophagy is harnessed in cancer treatment, and current clinical trials show promising results for melanoma, refractory myeloma, advanced solid tumors, and glioblastoma when chloroquine is combined with chemotherapy or RT [31]. These studies rely on treatment with chloroquine in combination with genotoxic anticancer drugs. However, little is known on the senolytic capacity of chloroquine. A recent study demonstrated that chloroquine acts as a senolytic agent in human fibroblasts [32]. To check this in our glioblastoma model, we treated senescent LN229 and A172 cells with chloroquine at a concentration non-toxic for proliferating cells (20 µM). Chloroquine treatment almost completely shifted cells from the senescent state to apoptosis in LN229 cells. Surprisingly, this effect was not seen when senescent A172 cells were treated. This marked difference might be due to differences in the pharmacokinetics (uptake) and/or genetic differences regulating CSEN between the lines. Thus, LN229 cells lack p16 and p14 while A172 cells express these proteins. Moreover, LN229 cells are PTEN competent, while A172 cells are PTEN deleted. The finding that senescent LN229, but not A172 cells are pushed into apoptosis following chloroquine treatment indicates that the response might depend on PTEN, which has been linked to autophagy regulation in glioma cells [33]. This example indicates that the effect of a senolytic agent is strongly dependent on the genetic constitution of the cells in question. 

A small molecule drug PX-866 targeting PI3K was shown to inhibit TMZ-induced autophagy [34]. This semisynthetic viridine was further intensively investigated for its anticancer properties, and promising results were obtained with glioma and MCF-7 breast cancer cells in vitro as well as in tumor xenografts when combined with TMZ, cisplatin, raloxifene or administered alone [34,35,36,37]. To investigate the PI3K inhibitor as to its senolytic activity in GBM cells, we treated senescent LN229 and A172 cells with low doses of PX-866 that were non-toxic on proliferating cells. After 2 d of exposure, we determined a significant decrease in the senescent population in both cell lines. Thus, PX-866 appears to exhibit senolytic activity in GBM cells. Another established inhibitor of autophagy is 3-MeA [38], which was, however, without senolytic activity in our assay. 

### 4.4. Agents Targeting DDR and DNA Repair

Triggered by the TMZ-induced primary damage O^6^MeG, the activation of DDR involving the ATM-ATR-Chk1-Chk2-p53 axis is required for the induction of cell death and CSEN [12,13]. However, little is known about the pathways involved in maintaining the senescent state. To elucidate the role of ATM/ATR in maintaining CSEN in GBM, we inhibited either one in senescent LN229 and A172 cells and measured senescence and apoptosis two days later. Inhibition of ATM or ATR led to a significant decrease in the senescent population, which was accompanied by an increase in apoptosis. Obviously, sustained ATM and ATR activation is essential for CSEN maintenance. This is in line with the findings that following TMZ treatment the O^6^MeG triggered DNA damage activates both ATR and ATM [39], and senescent GBM cells exhibit a sustained high level of DSBs [13]. ATM was also found to be required for maintaining the senescent state in HeLa cells [40]. 

We also tested inhibitors of Chk1, Chk2, p53 (pifithrin-a and µ) and p21, which however exhibited no senolytic activity. We should note that during the initiation phase of senescence, the Chk1/2-p53-p21 axis becomes activated, leading to a G2/M arrest [14]. Whether this pathway is required for maintenance of the senescent state in GBM cells is unclear. An alternative pathway may involve Wee1, which is also linked to the induction of a G2/M arrest in GBM cells [41]. Since Chk1 and Chk2 activate downstream overlapping pathways, we cannot exclude the possibility that their simultaneous inhibition causes an effect.

Another pathway shown to be involved in the induction of senescence rests on activation of the ATM-p62-NF-kB axis. In human fibroblasts ATM blocks p62-dependent degradation of GATA4 upon DNA damage, which is then able to activate NF-kB [42]. Actually, this pathway becomes activated following TMZ treatment in GBM cells [14]. Using the NF-kB inhibitor III, we observed an increase in apoptosis (presumably due to death of residual non-senescent cells), but not a reduction in the senescent population (as measured by C_12_FDG). Therefore, we do not consider the NF-kB inhibitor as a senolytic agent. 

We further tested the HR inhibitor Ri-1 [43] and the PARP1 inhibitor veliparib [44]. Both inhibitors were not effective as senolytics in our assays.

### 4.5. MCOPPB, Radiation and CCNU

A recent screening study identified MCOPPB, a compound originally studied for its potential anxiolytic activity, as a senolytic agent in human fibroblasts [45]. We tested the senolytic activity in our glioma cell model. Treatment of senescent LN229 and A172 cells with MCOPPB resulted in only very mild reduction of senescence and no increase in apoptosis. Thus, we do not consider MCOPPB a senolytic in our glioma cell system.

In the clinical setup, TMZ is combined with radiation therapy (RT) [46], leading to the question of whether RT is senolytic in glioma cells. Therefore, we irradiated senescent LN229 and A172 cells with increasing doses of γ-radiation. The data show that the senescence level was not significantly reduced with doses of up to 5 Gy, which were cytotoxic for proliferating cells. We therefore conclude that RT does not cause a senolytic effect on GBM cells. 

The DNA crosslinking chloroethylating agent CCNU (lomustine) is being used, often 2nd line, in glioma therapy [47] and effective when combined with TMZ [48]. In our assay, CCNU treatment of senescent LN229 and A172 cells did not result in a reduction of senescence in the population. Therefore, we do not consider CCNU to bear senolytic activity.

### 4.6. Natural Compounds

Three natural compounds were tested: fisetin, artesunate and curcumin. Fisetin, a polyphenol present in several fruits and vegetables and used as food supplement, has been found to be cytotoxic (at high dose) in several types of cancer, including glioma [49,50]. In an elegant series of experiments, it was also shown to reduce the amount of age-related senescent cells, using both in vitro and in vivo experimental systems [51]. To our best knowledge, fisetin has not yet been tested for its senolytic activity in glioma cells. Therefore, we treated TMZ-induced senescent LN229 and A172 cells with fisetin with a concentration non-toxic for proliferating cells. After 2 d of exposure, a significant reduction in senescence was observed, which was paralleled by an increase in apoptosis. We therefore consider fisetin to be a potent senolytic agent in GBM cells.

Artesunate is a semisynthetic derivate of artemisinin, a natural sesquiterpene lactone extracted from *Artemisia annua*. It has a long tradition in Chinese medicine (TCM) and, nowadays, is being used for the treatment of malaria [52]. Artesunate induces DNA damage and apoptosis in cancer cells, including GBM cells [53,54] and was shown to inhibit homologous recombination, thus sensitizing GBM cells to TMZ [55]. There are several trials going on with artesunate in cancer therapy, and reports indicate a beneficial role in the treatment of colon cancer and hepatocellular carcinoma [56,57,58]. Several studies suggest that artesunate not only induces apoptosis, but also CSEN [59,60]. Whether it also bears senolytic activity is unknown. To fill in this blank, we treated senescent LN229 and A172 cells with artesunate and found a significant decrease in CSEN accompanied by an increase in apoptosis. Since concentrations that were non-toxic on proliferating cells were used, the effect does not result from unspecific cytotoxicity but is, like ABT-737, CSEN specific. Therefore, artesunate can clearly be considered a senolytic agent in our GBM cell system. 

Another natural compound tested was curcumin. The ingredient of *Curcuma longa* is long known in TCM and used to treat a variety of inflammation-related diseases [61]. Lately, a number of studies have been performed on curcumin, proving its beneficial role in brain-related diseases and cancer [62,63,64,65]. Studies have also been performed on curcumin as an inducer of senescence [66,67] and senolytic agent [68]. In the experimental setting reported here curcumin at a subtoxic dose level neither reduced the senescent population nor induced apoptosis in senescent cells. Therefore, we do not consider curcumin as a senolytic agent in our GBM cell system. Nevertheless, curcumin might still pose a beneficial option for treatment of GBM since studies indicate that high-dose curcumin is effective alone [68] and when combined with RT and anticancer drugs [69,70,71,72]. 

## 5. Conclusions

From the inhibitors/compounds tested, the following had a specific impact on the survival of TMZ-induced senescent GBM cells and, therefore, can be considered to bear senolytic activity: ABT-737, navitoclax, chloroquine, ATMi, ATRi, BV-6, PX-866 and the natural compounds fisetin and artesunate. The senolytic capacity of fisetin and artesunate is of particular interest, as both natural compounds are widely used as food additive and also (at high concentration levels) therapeutically. In view of the lack of adverse effects, fisetin and artesunate might seriously be considered as adjuvant in glioma therapy. New delivery methods, such as encapsulation in lipids and micelles, are being developed to circumvent the low water solubility of fisetin and to make it useful for local administration [50]. The same holds true for artesunate, which is widely used for malaria treatment and considered safe. It should be noted that artesunate and fisetin at high dose levels are genotoxic and cytotoxic for cancer cells, including GBM ([55] and unpublished data) and therefore may exert both a tumor inhibiting and senolytic effect. Applied in supportive therapy for glioblastoma, artesunate proved to be tolerable if administered together with TMZ (H. Strik, person. communication). Since the primary therapy with IR and TMZ has a high senescence-inducing potential, the sequential use of senolytics deserves special attention in preventing tumor recurrence. 

## Data Availability

Data can be obtained by request from the corresponding author.

## Supplementary Materials

The following supporting information can be downloaded at: https://www.mdpi.com/article/10.3390/cells11162588/s1, Figure S1: Exemplary pictures of proliferating and senescent GMB cells. Figure S2: Toxicity testing on proliferating cells. Figure S3: Identification of small compounds with senolytic activity.
