# Supplementary material for: Abrogation of Cellular Senescence Induced by Temozolomide in Glioblastoma Cells: Search for Senolytics
Source: Cells. 2022 Aug 19;11(16):2588. doi: 10.3390/cells11162588 (PMC9406955; doi:10.3390/cells11162588)
Supplement: Supplementary file 1 [file cells-11-02588-s001.zip › cells-1861459-supplementary.pdf]

Abrogation of cellular senescence induced by temozolomide in glioblastoma cells: search for senolytics

Lea Beltzig, Markus Christmann, Bernd Kaina*

Institute of Toxicology, University Medical Center Mainz, Obere Zahlbacher Str. 67, D-55131 Mainz, Germany

Supplementary material

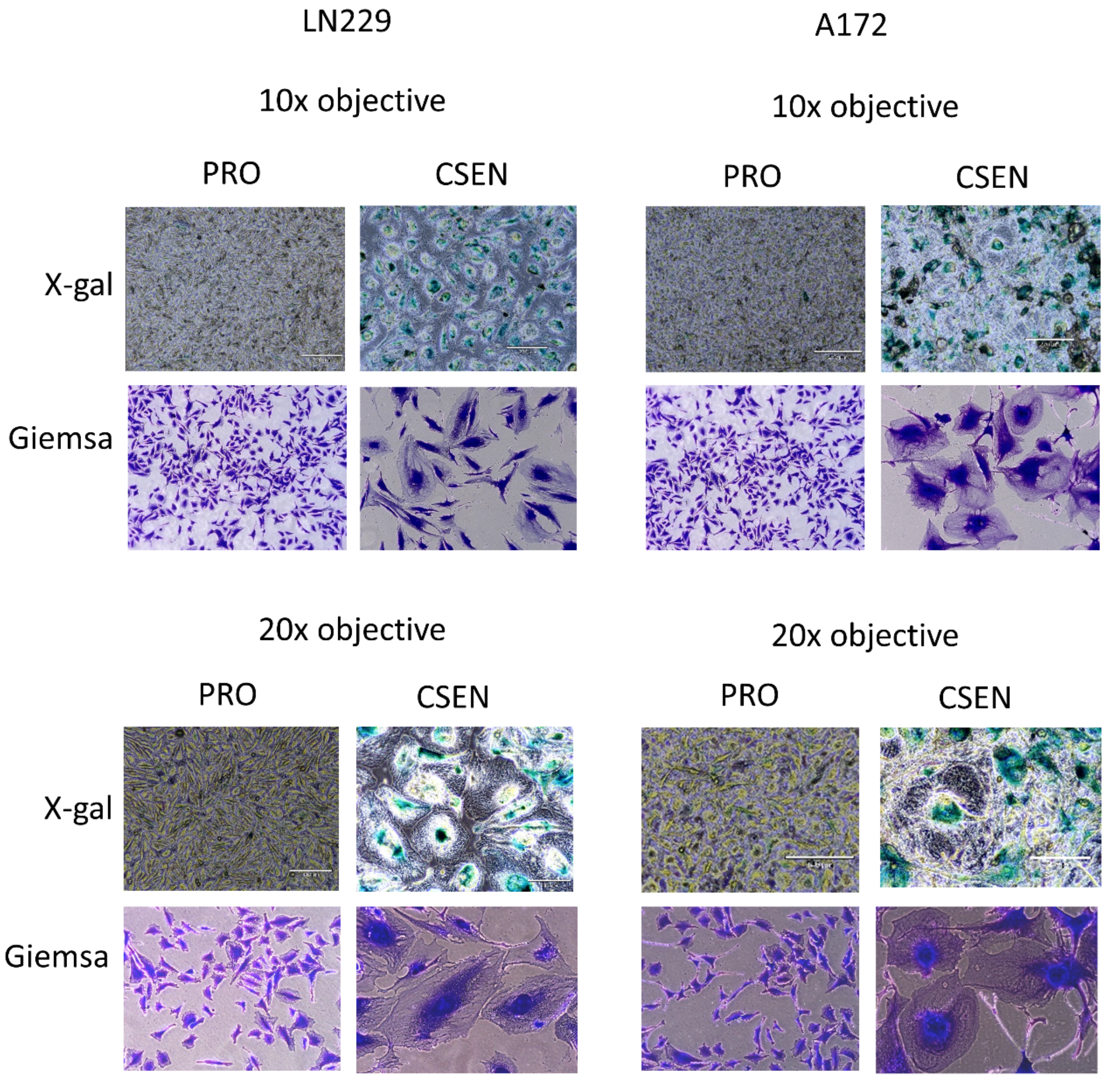

Figure S1. Exemplary pictures of proliferating and senescent GMB cells. Proliferating and senescent LN229 and A172 cells have been stained with X-gal or Giemsa. Photos were taken using a light microscope and a 10x objective (measuring bar represents 270 µm) or a 20x objective (measuring bar represents 140 µm).

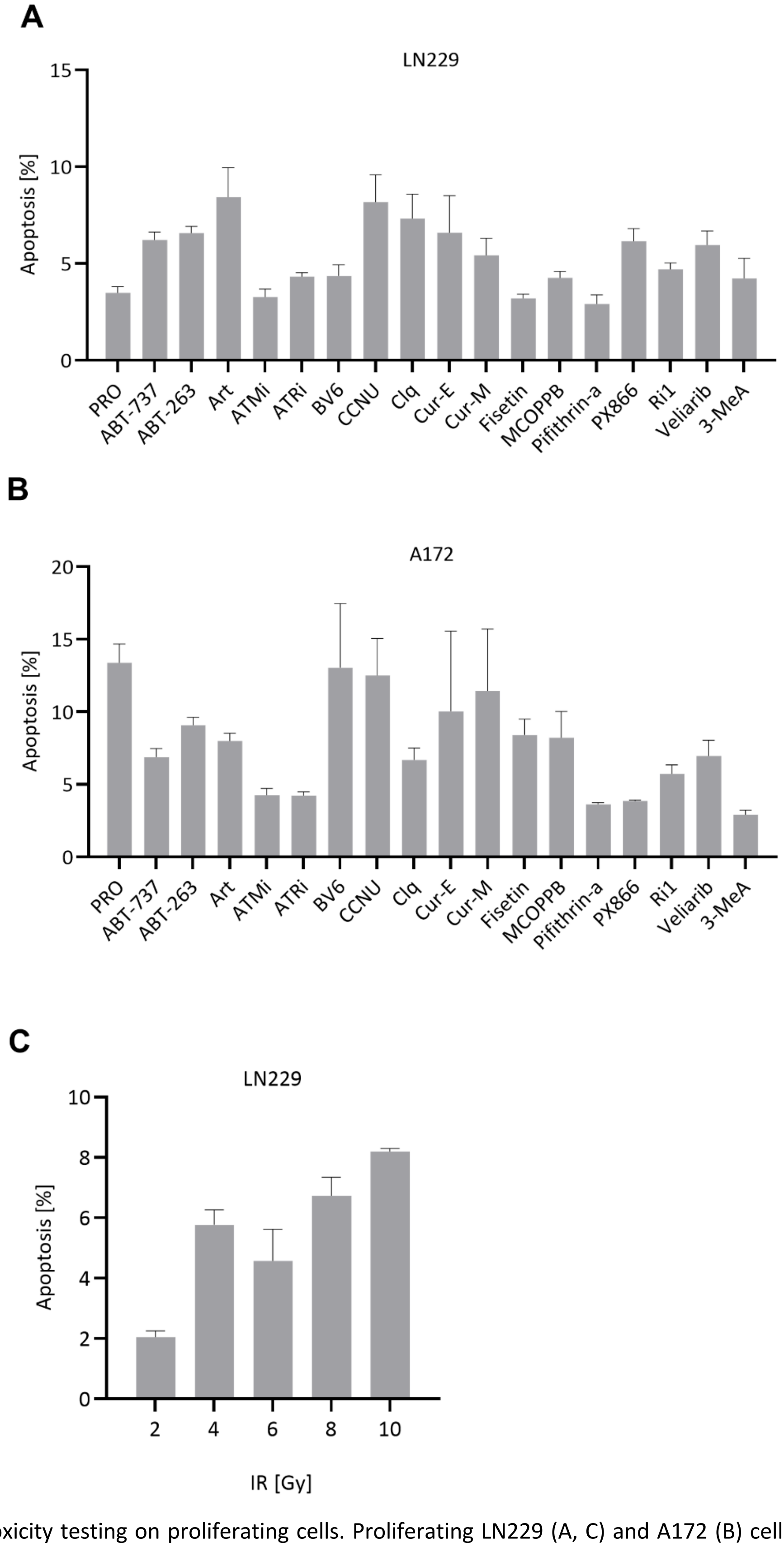

Figure S2. Toxicity testing on proliferating cells. Proliferating LN229 (A, C) and A172 (B) cells were treated with the indicated compounds (A, B) and ionizing radiation (IR) (C) to test for their cytotoxicity on proliferating cells. Non-toxic concentrations were used on senescent cells to test for senolytic activity. Data represent mean of >3 independent experiments ± SEM.

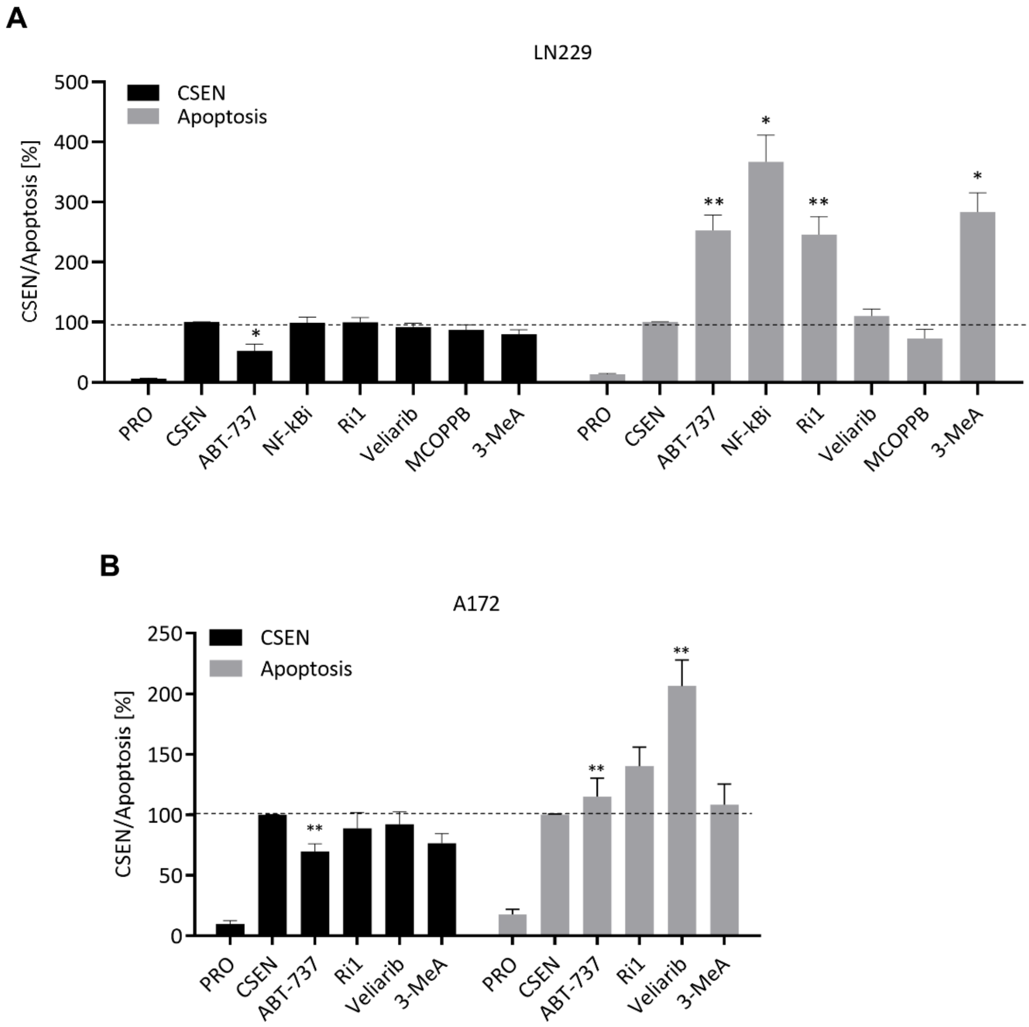

Figure S3. Identification of small compounds with senolytic activity. Proliferating (PRO) and senescent (CSEN) LN229 and A172 cells were treated with several substances to test for their senolytic capacity. ABT-737 served as positive control. Inhibition of NF-kB, Rad51 (Ri1), PARP (veliparib), the nociception receptor (MCOPPB) as well as inhibition of autophagy by 3-MA had no senolytic effect in LN229 (A) and A172 (B) cells. Data represent mean of >3 independent experiments ± SEM. *p<0.05, **p<0.01.
